# O uso de alimentos ultraprocessados como fonte de micronutrientes para crianças: risco de toxicidade?

**DOI:** 10.1590/0102-311XPT203323

**Published:** 2024-01-08

**Authors:** Márcia Regina Vítolo, Paola Seffrin Baratto, Caroline Nicola Sangalli, Paula dos Santos Leffa, Julia Luzzi Valmorbida

**Affiliations:** 1 Universidade Federal de Ciências da Saúde de Porto Alegre, Porto Alegre, Brasil.

Prezadas Editoras,

O artigo intitulado *Factors Associated with Anemia and Vitamin A Deficiency in Brazilian Children Under 5 Years Old: Brazilian National Survey on Child Nutrition (ENANI-2019)*^1^, publicado recentemente em CSP, revela dados que poderão ser utilizados na vigilância alimentar e nutricional para elaborar políticas públicas pertinentes à realidade nutricional das crianças brasileiras. O estudo avaliou 7.716 crianças entre 6 e 59 meses de idade, apresentando os fatores associados à anemia (concentração de hemoglobina < 11mg/dL) e à deficiência de vitamina A (nível de retinol sérico < 0,7μmol/L), por meio de análise hierárquica baseada no modelo teórico proposto pelo Fundo das Nações Unidas para a Infância (UNICEF). Entre todos os resultados apresentados por Castro et al. ^1^, destacam-se as baixas prevalências de anemia e vitamina A e sua associação com os chamados determinantes imediatos, como a ausência de aleitamento materno no dia anterior à entrevista em crianças de 6-23 meses e o consumo de 1-2 grupos de alimentos ultraprocessados no dia anterior em crianças de 24-59 meses de idade.

Visando colaborar com a reflexão sobre o tema, ressaltamos os resultados da publicação de Sangalli et al. ^2^, no qual foram encontradas baixas prevalências de inadequação de consumo de micronutrientes em crianças menores de cinco anos de baixa condição socioeconômica. À época, esses resultados nos surpreenderam, pois, se a qualidade da alimentação era ruim, de onde estavam vindo os micronutrientes consumidos por essas crianças? Valmorbida et al. ^3^ haviam demonstrado, em publicação anterior, com a mesma amostra de crianças, que 58% e 87,4% não consumiram sequer uma porção de frutas e verduras, respectivamente. Na publicação de Sangalli et al. ^2^, a inclusão da informação nutricional contida no rótulo das embalagens no cálculo nutricional dos inquéritos recordatórios de 24 horas mostrou que os alimentos ultraprocessados forneciam a maior porcentagem de micronutrientes presentes nas dietas. Além da alta densidade de açúcar, gordura, sódio e aditivos, alimentos ultraprocessados são adicionados de micronutrientes para, entre outras finalidades, atrair compradores com as alegações de que são importantes fontes de ferro, vitamina C, vitamina A e zinco. A presença de ultraprocessados enriquecidos com micronutrientes (achocolatados, biscoitos, cereais para crianças, suco artificial, gelatinas) era predominante nos inquéritos alimentares das crianças (88,1%). O percentual de contribuição de micronutrientes fornecidos por esses alimentos foi de: 38,3% para ferro; 20,4% para vitamina A; 19,7% para cálcio; 17,2% para vitamina C; 14,6% para zinco; e 11,3% para folato. O resultado mais preocupante foi a ingestão de micronutrientes acima da *upper level*, que é o nível máximo de segurança de ingestão para prevenir toxicidade. Para a vitamina A, 4% das crianças ultrapassaram a *upper level*; para o zinco, 3,1%; para ácido fólico, 1,1%; e para o ferro, 0,2%.

Ainda que os métodos de análise de consumo alimentar de ultraprocessados tenham sido diferentes entre o estudo de Castro et al. ^1^ (consumo no dia anterior, sim/não) e o de Sangalli et al. ^2^ (inquérito recordatório de 24 horas), ambos os resultados corroboram outros estudos que sugerem que a ingestão excessiva de micronutrientes por crianças tem ocorrido devido à prática de suplementações medicamentosas ou dietéticas, fortificações ou enriquecimentos de alimentos. Dois estudos realizados nos Estados Unidos mostraram que os alimentos enriquecidos forma as principais fontes de micronutrientes em crianças e adolescentes ^4,^ ^5^, evidenciando prevalências significativas de crianças com ingestão de sódio, vitamina A e zinco acima da *upper level*.

É importante traçar um paralelo entre a fortificação das farinhas de milho e trigo com ferro e ácido fólico, que é mandatário no Brasil, e a adição de micronutrientes nos alimentos ultraprocessados, realizada de forma voluntária e estratégica pela indústria alimentícia. A fortificação das farinhas não implica risco para consumo excessivo, uma vez que a quantidade de 4 a 9mg de ferro e 150 a 220mcg de ácido fólico por 100g de farinha não impacta significativamente no consumo diário total ^6^. Além disso, estudos de análise temporal demonstram que preparações culinárias que utilizam essas farinhas estão sendo gradualmente substituídas por alimentos ultraprocessados ^7^^,^^8^, que contribuem com mais da metade da ingestão energética diária de crianças americanas e europeias e com cerca de um terço das calorias ingeridas por crianças de países em desenvolvimento ^9^. Nesse cenário, entendemos que os alimentos ultraprocessados atuam como principais veículos dos micronutrientes sintéticos consumidos por crianças de diferentes faixas etárias.

Mesmo a situação de saúde no Brasil sendo de tripla carga de má nutrição infantil (coexistência de desnutrição, deficiências de micronutrientes e excesso de peso) e a fortificação de alimentos sendo uma das possíveis estratégias de prevenção de deficiência, a adição de micronutrientes motivada por apelo comercial pode favorecer desfechos negativos na saúde. Isso porque os micronutrientes mais comumente adicionados (p.ex.: ferro, vitamina A e zinco) são, muitas vezes, os mesmos fornecidos nas estratégias nacionais de fortificação da alimentação infantil. A coexistência de múltiplas intervenções para resolver deficiências de micronutrientes é preocupante ^10^, principalmente pelo consumo excessivo de ferro, que pode causar diarreia (mediada pela diminuição de bifidobactérias e aumento de enterobactérias, o que favorece a proliferação de patógenos oportunistas, como *Escherichia coli*), e aumentar o risco de morbidade gastrointestinal ^11^. O excesso de vitaminas de diferentes classes também parece exercer efeito no desenvolvimento de obesidade ^12^.

Assim, é de extrema importância que futuros estudos considerem que: (1) a informação nutricional dos alimentos ultraprocessados precisa ser incluída na investigação dietética e no cálculo nutricional; (2) a presença de alimentos ultraprocessados pode ser mais prevalente em populações de baixa condição socioeconômica, uma vez que esses alimentos têm menor custo e são, portanto, mais acessíveis; e (3) as crianças com alta ingestão de alimentos ultraprocessados estão em risco de apresentar toxicidade por consumo excessivo de micronutrientes, considerando que esta só é observada por meio de micronutrientes sintéticos adicionados aos alimentos ou suplementos.
